# Accuracy of Cone-Beam Computed Tomography in Determining the Root Canal Morphology of Mandibular First Molars

**DOI:** 10.7508/iej.2016.02.005

**Published:** 2016-03-20

**Authors:** Hadi Mokhtari, Mahdi Niknami, Hamid Reza Mokhtari Zonouzi, Aydin Sohrabi, Negin Ghasemi, Amir Akbari Golzar

**Affiliations:** a * Dental and Periodontal Research Center, Department of Endodontics, Dental School, Tabriz University of Medical Sciences, Tabriz, Iran; *; b * Department of Oral and Maxillofacial Radiology, Dental School, Tehran University of Medical Sciences, Tehran, Iran; *; c *Department of Orthodontics, Dental School, Tabriz University of Medical Sciences, Tabriz, Iran; *; d * Private Practitioner, Tabriz, Iran*

**Keywords:** Cone-Beam Computed Tomography, Root Canal Morphology, Root Clearing

## Abstract

**Introduction:**

The aim of the present *in vitro *study was to compare the accuracy of cone-beam computed tomography (CBCT) in determining root canal morphology of mandibular first molars in comparison with staining and clearing technique.

**Methods and Materials:**

CBCT images were taken from 96 extracted human mandibular first molars and the teeth were then evaluated based on Vertucci’s classification to determine the root canal morphology. Afterwards, access cavities were prepared and India ink was injected into the canals with an insulin syringe. The teeth were demineralized with 5% nitric acid. Finally, the cleared teeth were evaluated under a magnifying glass at 5× magnification to determine the root canal morphology. Data were analyzed using the SPSS software. The Fisher’s exact test assessed the differences between the mesial and distal canals and the Cohen’s kappa test was used to assess the level of agreement between the methods. Statistical significance was defined at 0.05.

**Results:**

The Kappa coefficient for agreement between the two methods evaluating canal types was 0.346 (95% CI: 0.247-0.445), which is considered a fair level of agreement based on classification of Koch and Landis. The agreement between CBCT and Vertucci’s classification was 52.6% (95% CI: 45.54-59.66%), with a significantly higher agreement rate in the mesial canals (28.1%) compared to the distal canals (77.1%) (*P*<0.001).

**Conclusion:**

Under the limitations of this study, clearing technique was more accurate than CBCT in providing accurate picture of the root canal anatomy of mandibular first molars.

## Introduction

Successful root canal treatment depends on thorough cleaning of all the canals in the root canal system and proper obturation of the canals to achieve a fluid tight seal [1]. Therefore, proper knowledge about the morphology of the root canal system is an important prerequisite for endodontic treatment and lack of sufficient knowledge about root canal anatomy might result in missing the root canals and subsequent treatment failure [2, 3]. Therefore, considering the importance of such knowledge, it is necessary to determine a standard *in vitro* method to accurately determine the morphology and position of the root canals. 

Routine and commonly used techniques to evaluate the root canal morphology include staining and clearing [4], conventional radiography [5-7], digital radiography [8, 9], radiography with contrast media [10, 11] and finally, computed tomography which has been introduced to this end in recent years [12-15]. Among these techniques, staining and clearing is the most commonly used technique and is considered as the gold standard to determine the root canal morphology [16, 17]. However, this technique is destructive and highly technique sensitive, requires sectioning or clearing of the teeth for the evaluation of the root canal morphology and cannot be employed in clinical situations when radiography cannot show the complex anatomy of the root canal system and the dentist needs to know the presence of accessory root canals, *etc.* [18]. 

**Figure 1:**
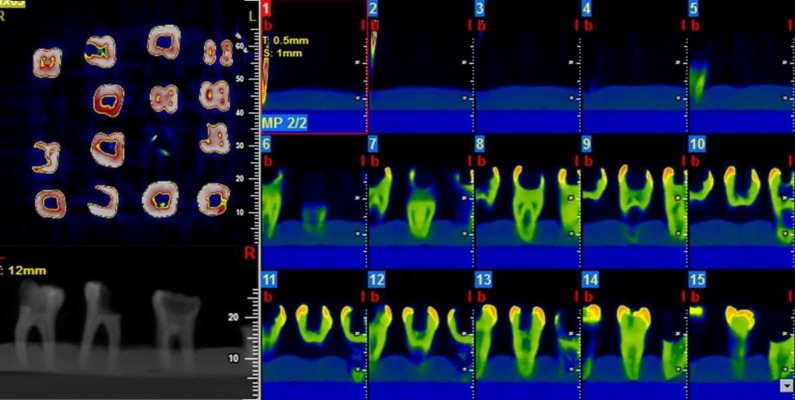
Volume-rendering and multi-planar volume reconstruction of images using the NTT viewer software program at 0.5-mm-thick coronal cross-sections

**Figure 2:**
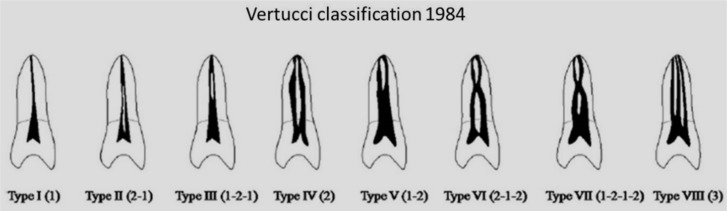
Vertucci’s classification of root canal morphology

In addition, conventional and digital radiographies are two-dimensional (2D) representations of three-dimensional (3D) structures; therefore, they are not accurate in determining the root canal morphology. A new technique for the evaluation of root canal morphology is cone-beam computed tomography (CBCT) which can reconstruct the three-dimensional structure of the root canal system to provide the dental practitioner with an accurate image of root canal [19-21]. This technique is simple and non-destructive and can be carried out in clinical settings. The advantages of CBCT include its non-destructive nature, three-dimensional reconstruction of the root canal system, a decrease in x-ray dose, the feasibility of limiting the imaging field (which also decreases the x-ray dose), an increase in image accuracy and resolution and a decrease in imaging errors such as artifacts [22, 23].

The first posterior teeth to erupt into the oral cavity are mandibular first molars, which generally require extensive restorations due to a high incidence rate of caries and the need for endodontic treatment [24]. On the other hand, considering the effect of ethnic differences on the type and number of root canals it is important to introduce a technique with the highest accuracy, minimum destruction and the possibility for using in clinical situations. 

In recent years, CBCT has been the subject of many studies in different endodontic procedures, including evaluation of the internal morphology of the root canal system [18, 25]. The aim of the present *in vitro* study was to evaluate the accuracy of CBCT technique in determining the morphology of the root canal system of mandibular first molars in comparison with the staining and clearing technique.

## Materials and Methods

***Selection and preparation of samples***

A total of 96 human mandibular first molars extracted for periodontal reasons were evaluated. The teeth had mature apices and uncalcified root canals judged by initial radiographic examination. Soft tissue remnants were removed from the root surfaces and the samples were stored in 0.5% chloramine-T solution until being used for the study. All the existing metallic restorations were eliminated and the teeth were randomly inserted into foam arches in close contact with each other to simulate their natural alignment in a dental arch in 6 groups (*n*=16). 

***Evaluation of the root canal system anatomy with CBCT***

CBCT images were prepared in high-resolution zoom mode with voxel size of 0.15 mm using NewTom VGi 9000 CBCT device (Quantitative Radiology SRL Co., Verona, Italy) with a 12×7.5 cm background using 110 kVp, 19 mA and 18 sec of exposure time. Volume-rendering and multi-planar reconstruction was performed using the NTT viewer software program (NTT Software Corporation, Yokohama, Japan) at 0.5 mm-thick coronal cross-sections. Then the 3D reconstructed images were evaluated by three endodontists based on Vertucci’s [17] classification (Figures 1 and 2) who were assigned to evaluate the configuration of mesial and distal canals. Each classification needed to be agreed upon by at least two endodontists. 

***Evaluation of the root canal system morphology by dye penetration and clearing***

After determination of each tooth morphology with CBCT, for each tooth an access cavity was prepared with a cylindrical fissure bur (D&Z, Diamant, Germany). Then the pulp chamber floor was explored with an endodontic explorer (Hu Freiday, Chicago, IL, USA) to locate the canal orifices. Canals were minimally instrumented with #10 file to facilitate the complete removal of pulp tissue and penetration of the dye inside the root canal. Then teeth were immersed in 5.25% NaOCl (Golrang, Tehran, Iran) solution for 48 h to eliminate pulp remnants and debris. Subsequently, India ink (Quink, Parker Pen Holding Ltd., Epping, England) was injected into the root canals with an insulin syringe. Then the teeth were demineralized at room temperature (20^°^C) with 5% nitric acid for 3 days. Fresh nitric acid was used each day. Then the teeth were rinsed in water for 4 h, followed by a dehydration process with 80% ethyl alcohol (Ararat, Tehran, Iran) for 24 h, 90% ethyl alcohol for 1 h and finally 100% ethyl alcohol for 1 h. The dehydrated teeth were then immersed in methyl salicylates (Merck, Darmstadt, Germany) for 2 h for clearing. Finally, the cleared teeth were evaluated by three endodontists under a magnifying glass (Lumagny, No. 7540, Hong Kong) under 5× magnification for determining the root canal morphology based on Vertucci’s classification. The classification that was agreed upon by at least two observers was accepted and recorded.

**Table 1:** Distribution of different canal types detected by CBCT and clearing techniques

**Vertucci’s classification**	**CBCT (N)**	**Clearing (N)**
Type I	46.9	44.3
Type II	49.5	13.5
Type III	1.6	10.9
Type IV	2.1	24.0
Type V	-	5.2
Type VI	-	1.0
Type VII	-	-
Type VIII	-	-

***Data analysis***

The data were analyzed with SPSS software (SPSS version 19.0, SPSS, Chicago, IL, USA), using descriptive statistical tests; the accuracy of CBCT technique was calculated in percentages. The Fisher’s exact test was used to evaluate the differences between mesial and distal canals. Inter-method agreement was evaluated by the Cohen’s kappa test. Statistical significance was set at 0.05.

## Results

In the present study, 96 mandibular first molars and 192 root canals were evaluated. Table 1 presents the distribution of root canal types based on Vertucci’s classification as determined by CBCT and clearing techniques. Table 2 presents the different types of root canals in the mesial and distal roots of mandibular first molars using the two aforementioned techniques.

Evaluation of inter-method agreement in relation to canal types showed a kappa coefficient of 0.346 (95% CI: 0.247-0.445), which is a fair level of agreement based on classification of Koch and Landis [26]. In 52.6% of cases (at 95% CI: 45.54-59.66%) the results of CBCT technique were consistent with Vertucci’s classification, with 28.1% in the mesial canals (at 95% CI: 19.11-27.09%) and 77.1% in the distal canals (at 95% CI: 68.69-85.51%), revealing statistically significant differences (*P*<0.001).

## Discussion

In the present study, the accuracy of CBCT technique for the evaluation of root canal morphology of mandibular first molars was compared with that of staining and clearing technique and the results showed lower validity of CBCT images in comparison with the gold standard. 

Cleaning of the entire root canal system is an important prerequisite for the success of root canal treatment. The root canal system anatomy is very complex and it is rather difficult to evaluate [27]. The calcified pathways of the root canal should be paved so that the morphology of the root canals can be visualized. Different techniques have been used for evaluation of the internal anatomy of teeth; for example, extracted teeth undergo radiography from different mesiodistal and buccolingual directions to produce 3D images. In another technique, India ink is injected into the root canal system using an insulin syringe and the internal anatomy of the teeth is examined after decalcification of cleared teeth. Tooth clearing techniques are considered as reliable techniques for the visualization of the entire root canal system [28]. This technique visualizes the lateral root canals, transverse anastomoses, apical deltas and other complexities of the root canal system. This technique was also used by Vertucci and other researchers can exactly reveal the internal anatomy and anastomoses in a 3D configuration but it cannot be used in the oral cavity and clinical settings [2].

**Table 2:** Distribution of different canal types in the mesial (M) and distal (D) roots of mandibular first molars detected by CBCT and clearing techniques

**Canal type**	**Type I**		**Type II**		**Type III**		**Type IV**		**Type V**		**Type VI**		**Type VII**		**Type VIII**	
	**M**	**D**	**M**	**D**	**M**	**D**	**M**	**D**	**M**	**D**	**M**	**D**	**M**	**D**	**M**	**D**
**Clearing**	2	61	6	8	3	6	76	13	6	4	6	-	-	-	-	-
**CBCT**	1	86	9	2	1	5	84	2	-	-	-	-	-	-	-	-

Evaluation of the tooth structure clinically by CBCT images is a new technique in endodontics [29]. 

The advent of CBCT technique has revolutionized the imaging techniques in the craniofacial complex. The most important property of the system that makes it useful from the endodontic point of view, is the ability to provide 3D images of the internal anatomy of root canals. With the use of CBCT it is possible to analyze the images with software programs without interfering with the main format of digital images (Digital Imaging and Communications in Medicine, DICOM), which makes it possible to evaluate the treatment success, canal transportation direction and 3D analysis of the canals [22, 23].

Various studies have reported different hypotheses and findings in relation to the anatomy of root canal systems, which might be attributed in part to differences in the internal anatomy of root canals and in part to problems encountered during the study of a root canal system and to the techniques and classification systems used by different researchers. In addition, another potential problem might be mistaking the mandibular second molar for neighboring first molars due to the great similarity between the anatomies of these teeth.

The completion of Vertucci’s classification in previous studies has been proved [24, 30]. This system was used in the present study as a reference for evaluation of the root canal configuration and its internal anatomy due to the accuracy of its procedural steps. In the study by Vertucci, injection of dye was carried out before clearing, which was followed in the present study, resulting in diffusion of dye. It appears that if injection of dye is carried out after clearing, better results can be achieved [31, 32]. Therefore, it is better to modify the dye injection and clearing procedures used by Vertucci in future studies. 

Mandibular first molars are particularly important in studies on root canal system anatomy due to a wide range of anatomic variations and differences in the statistics in relation to the number and morphology of root canals [16, 18, 33-35]. Studies have shown high prevalence of single canal in the distal roots of mandibular first molars [2], as in the present study. The prevalence of a single canal in the distal root has been reported to be 50 to 84% [24]. According to these studies, single canals were found in 72% [36] and 81% [37] of the distal roots, which is similar to the results of the present study.

Indeed, the variations in the root canal type of the mesial roots in our study ranged from Vertucci’s type *I* to type *VI*. The same as the previous studies [30], type *IV* was the most frequent. Neelakantan *et al.* [18] reported similar results with CBCT and staining and clearing techniques in determining the morphology of the root canal system; however, in their study only 21 mandibular first molars were evaluated. Michetti *et al.* [25] reported the same results with the use of CBCT and staining and sectioning techniques; however, in that study clearing technique was not used, resulting in a decrease in the accuracy of the results. 

In the present study the clearing technique was more accurate than CBCT in determining the anatomy of the root canal system of mandibular first molars, which is contrary to the results of previous studies. Such a discrepancy in results might be attributed to differences in methodologies, including the sample size, the type of CBCT device and tooth type. In addition, the inherently low contrast resolution of the CBCT technique should be mentioned, which under the best conditions, is considered a negative factor in the diagnostic quality of images and even increases with an increase in image size.

## Conclusion

Considering the limitations of the present study, the clearing technique was more accurate than CBCT in discovering the internal anatomy of mandibular first molars.
